# Influence of Immersion Orientation on Microstructural Evolution and Deformation Behavior of 40Cr Steel Automobile Front Axle during Oil Quenching

**DOI:** 10.3390/ma17184654

**Published:** 2024-09-23

**Authors:** Yuanji Shi, Xiaowen Wang, Chengtong Dong, Junwan Li, Zeyu Chen, Cheng Cheng

**Affiliations:** 1 School of Mechanical Engineering, Nantong Institute of Technology, Nantong 226005, China; shiyj@niit.edu.cn; 2 Industrial Perception and Intelligent Manufacturing Equipment Engineering Research Center of Jiangsu Province, Nanjing Vocational University of Industry Technology, Nanjing 210023, China; 13092301728@163.com; 3 Key Laboratory of Advanced Technology for Automotive Components, Wuhan University of Technology, Wuhan 430070, China; xiaowen_wang24@163.com; 4 School of Materials Science and Engineering, Shanghai University, Shanghai 200444, China; dongct1997@126.com; 5 College of Material Science and Technology, Nanjing University of Aeronautics and Astronautics, Nanjing 210016, China; c_cheng@nuaa.edu.cn

**Keywords:** front axle, quenching, phase transformation, deformation behavior

## Abstract

This study employs the finite element method to investigate the microstructural evolution and deformation behavior of a 40Cr steel automobile front axle during the quenching process. By establishing a multi-physics field coupling model, the study elucidates the variation patterns of the microstructure field in the quenching process of the front axle under different immersion orientations. It is found that along the length direction, the bainite and martensite structures decrease from the center to the edge region, while the ferrite structure shows an increasing trend. Additionally, the influence of immersion orientation on the hardness of the front axle’s microstructure and deformation behavior is thoroughly discussed. The results indicate that, firstly, when quenched horizontally, the hardness difference among different regions of the front axle is approximately 8.2 HRC, whereas it reaches 10.3 HRC when quenched vertically. Considering the uniformity of the microstructure, the horizontal immersion method is preferable. Secondly, due to the different immersion sequences in different regions of the front axle leading to varying heat transfer rates, as well as the different amounts of martensite structures obtained in different regions, the deformation decreases along the length direction from the center to the edge region. Horizontal immersion quenching, compared to vertical immersion, results in a reduction of approximately 56.2% and 48.9% in deformation on the representative central cross-section (A-A) and the total length of the front axle, respectively. Therefore, considering aspects such as microstructure uniformity and deformation, the horizontal immersion quenching orientation is more favorable.

## 1. Introduction

The automobile front axle, as a crucial safety component, is typically made of 40Cr (AISI 5140) steel. It operates under harsh and complex conditions, bearing predominantly alternating loads. Ensuring that it remains fault-free under operational loads is essential [1]. Hence, higher demands are placed on the performance and manufacturing processes of the front axle [2]. The quenching process is one of the key steps to enhance the primary performance of the front axle. The choice of immersion orientations during the quenching process significantly impacts the material’s microstructural evolution, stress development, and other performance aspects of the automobile front axle [3]. Choosing the appropriate immersion methods can effectively optimize the quenching process of the automobile front axle, improving hardness, uneven temperature distribution during cooling, and enhancing the stability of the front axle dimensions [4].

In recent years, a significant number of scholars have systematically studied and analyzed the stress distribution, deformation behavior, and other aspects of automobile components using finite element analysis, aiming to obtain more reliable data support. Avikal [5] performed 3D modeling of the front axle beam of automobiles and conducted fatigue life analysis using ANSYS 2022R1 software. The study revealed that the maximum stress occurs on the surface at the end region where the beam connects to the wheel, and cracks originate near the surface. Further analysis was carried out on various parameters such as deformation and fatigue life for materials including 50 steel, AISI 1045, AISI 4130, AISI 4140, and AISI 4150. It was found that 50 steel exhibits the least deformation, while the axle beam made of AISI 4150 material possesses the maximum fatigue life. Sathish [6] conducted a study focusing on the analysis of automobile connecting rods made of three different aluminum alloy materials. Finite element analysis was conducted using ANSYS software to obtain von Mises stress, shear stress, and total deformation. The results indicated that AA2014 exhibits lighter weight and better stiffness, with a reduction in deformation of approximately 17% and 13% compared to A6061 and AA7075, respectively. M.M. Topaç [7] explored the fatigue failure causes of steel wheels in vehicles through finite element analysis, identified stress concentration regions, and employed a design enhancement solution. This solution involved increasing the local thickness (*t_c_*) and the cross-sectional radius (*r_c_*) in critical areas to reduce the equivalent von Mises stress in these regions. Witek [8] established geometric models for the connecting rod, piston, and adjacent components. Through finite element analysis, it was discovered that cracks originated in the region of maximum principal stress, near the bolt hole of the connecting rod, with stress values reaching 62% of the material yield strength. Seralathan [9] employed the finite element method to conduct analyses on the total deformation, equivalent elastic strain, and equivalent stress of the connecting rod in a compression ignition engine. The study revealed that, compared to the base material A356, the equivalent stress distribution of the A356-5%SiC-10% Flyash stir cum squeeze casting material was nearly similar, while the total deformation decreased by 38.50%.Afolabi [10] employed the finite element analysis method to analyze the plastic deformation of the shaft in a nut-breaking machine, aiming to prevent mechanical component failures. The results indicate that, under the maximum torque of 72.0 Nm, the safety factor for a shaft with a diameter of 20 mm is 2.0. This suggests that the shaft can withstand twice the load, while also adhering to the von Mises (VMS) theory, demonstrating good safety and economic viability.

It is noteworthy that, during heat treatment, 40Cr steel undergoes deformation due to thermal expansion and phase transformation during quenching. This deformation leads to dimensional changes in the workpiece, affecting the precision of assembly during the assembly process. Leblond [11] proposed a theoretical method for solving the phase transformation strain rate problem in the Magee mechanism. They provided an expression for the phase transformation plastic strain rate of steel under low-stress conditions. This expression is consistent with experimental results for various materials. Finite element simulations verified the theoretical formula, allowing a detailed examination of the effectiveness of some physical assumptions made in the treatment. It also permits the study of phase transformation plasticity under high stress, leading to the proposal of a general model applicable to various stress conditions during the ideal plastic stage. In the same year, Leblond [12] further refined the expression of the phase transformation plastic strain rate by appropriately modifying the processing method used in the first part. Finally, they provided a supplementary evolution equation for hardening parameters. Since deformation inevitably occurs during the phase transformation process, and martensite is the primary phase transformation phase during the quenching process of steel components, which significantly affects deformation, Gür [13] established and implemented an effective finite element model. This model predicts the evolution of the temperature field, phase volume fraction, and internal stresses towards the residual stress state during the quenching process of axisymmetric steel components. Canale [14] discussed the influence of various factors on quenching deformation and steel cracking. They elucidated the simulation methods for the quenching process and their potential significant benefits in system and process design. Additionally, they explored the application of computational fluid dynamics (CFD) analysis in quenching system design. Lee [15] investigated the relationship between phase transformation dynamics and deformation, proposing new martensite start temperatures and martensite kinetics equations. Simultaneously, related studies utilized finite element analysis methods to analyze and discuss deformation behavior. Greenwood [16] studied the deformation behavior under small stress during the phase transformation process in metals with various crystal structures. Li [17] employed a thermomechanically coupled rigid–plastic finite element method to simulate the roller forging process of the automobile front axle blank. The study analyzed the metal flow process in the rolling die, the equivalent stress–strain field, temperature field, and load state.

To further explore the impact of immersion methods on the quenching of automobile front axles and optimize the heat treatment process, this study, based on the metal–thermal–mechanical coupling theory framework, establishes a finite element model for the automobile front axle. Using numerical simulation, it quantitatively characterizes the evolution of microstructure and deformation behavior during the quenching of the 40Cr automobile front axle. The study analyzes the influence of immersion orientation on the microstructural evolution and deformation behavior of the automobile front axle during quenching, elucidates the mechanisms of various factors during the quenching process, and provides insights for the optimization of the quenching process for automobile front axles.

## 2. Experimental

**Figure 1:**
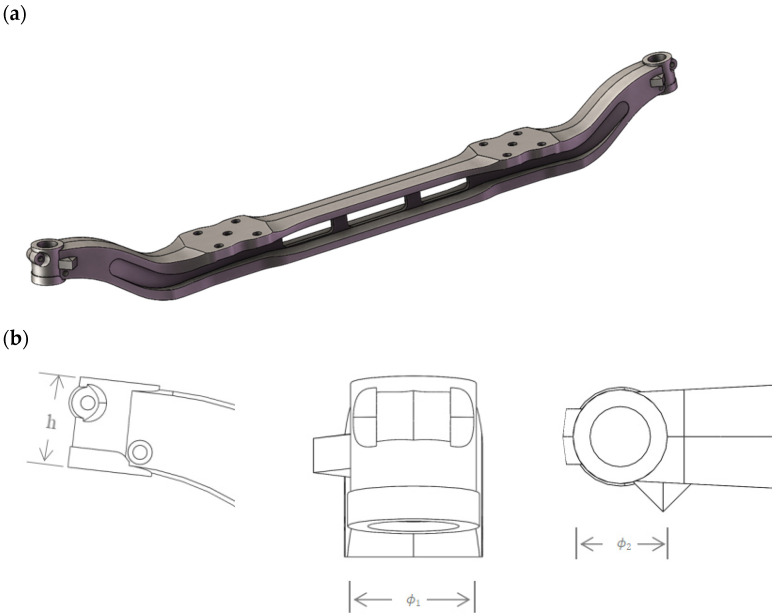
Automobile front axle and three views of axle head. (**a**) Three-dimensional solid representation. (**b**) Frontal view, lateral view, and superior view of the axis head.

**Figure 2:**
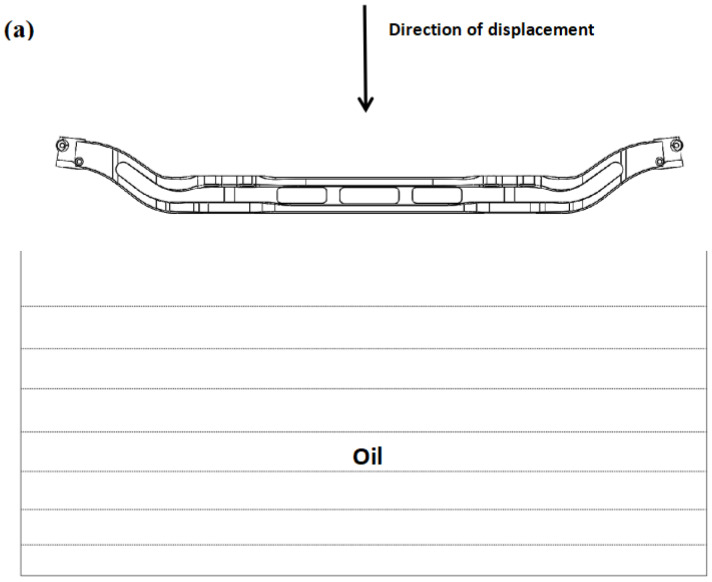

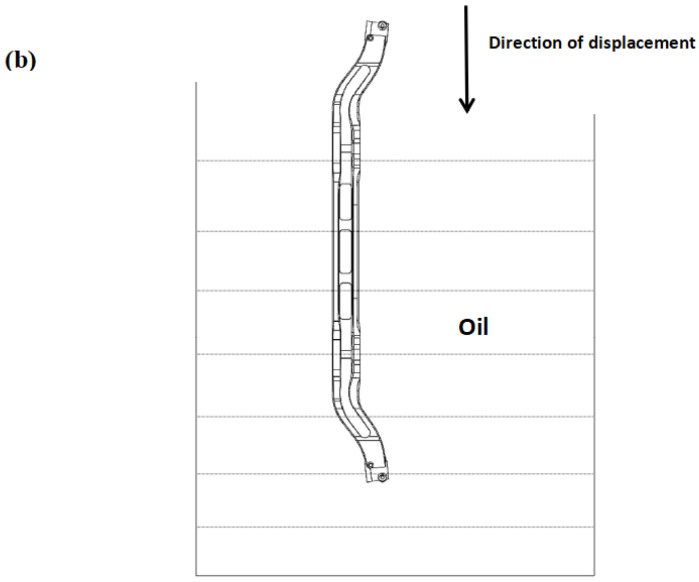
Schematic diagram of the immersion orientation during cooling: (**a**) horizontal immersion; (**b**) vertical immersion.

The parts used in the study are automobile front axles made of 40Cr steel, with chemical composition (nominal composition) as shown in Table 1. Figure 1 shows the three views of the automobile front axle and its axle head used in the heat treatment experiment. The total length of the front axle is 1487.06 mm, and the dimensions of the axle head are h = 70 mm, φ1 = φ2 = 52.23 mm. In the experiment, the automobile front axle is heated to 850 °C in the heat treatment furnace and held for 2.5 h. Subsequently, front axles with different batch numbers are quenched in the cooling medium to room temperature using different immersion methods. The quenching medium is oil, and the immersion orientations are horizontal immersion and vertical immersion, as shown in Figure 2. Figure 2a illustrates horizontal immersion into the quenching medium, where the bottom surface of the automobile front axle is parallel to the coolant’s horizontal plane, and rapidly immersed. Figure 2b depicts the vertical immersion into the quenching medium, where the bottom surface of the automobile front axle is perpendicular to the coolant’s horizontal plane and enters the oil at a speed of 0.15 m/s.

The Tango-S Plus handheld portable 3D scanner was used to scan the dimensions of the front axle before and after quenching. The scan results are processed into solid entities with accurate positioning. By comparing the dimensional data of the same sample before and after quenching, a deformation data analysis report is generated. After the deformation measurement is completed, the sections required for metallographic analysis were obtained through sawing and wire cutting. Hardness is measured using a 69-1 Rockwell hardness tester according to the standard GB/T 230.1-2018 [18] (with a loading force of 150 kg and loading time of 10 s). Further analysis is conducted using the EPIPHOT 300 metallographic microscope (Shanghai Changfang Optical Instrument Co., Ltd. in Shanghai, China). The samples obtained through wire cutting have dimensions of 10 mm × 10 mm × 15 mm. The metallographic preparation follows conventional methods, with a corrosion solution of 4% concentration nitric acid in alcohol and an corrosion time of approximately 3–5 s.

**Figure 3:**
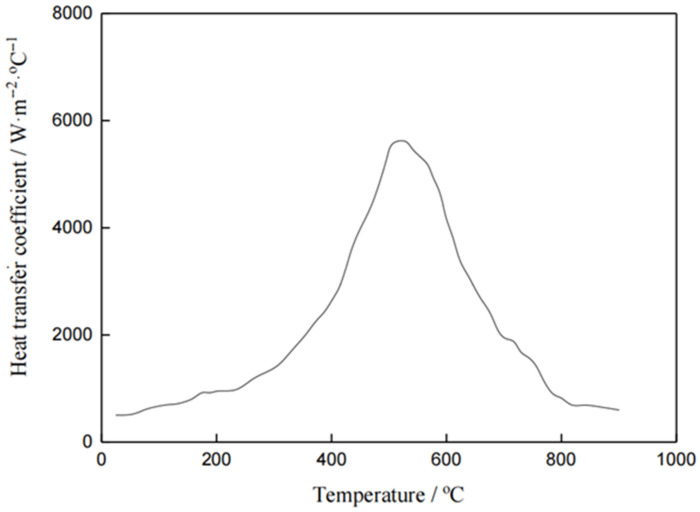
Heat transfer coefficient of the cooling medium (oil at room temperature).

**Figure 4:**
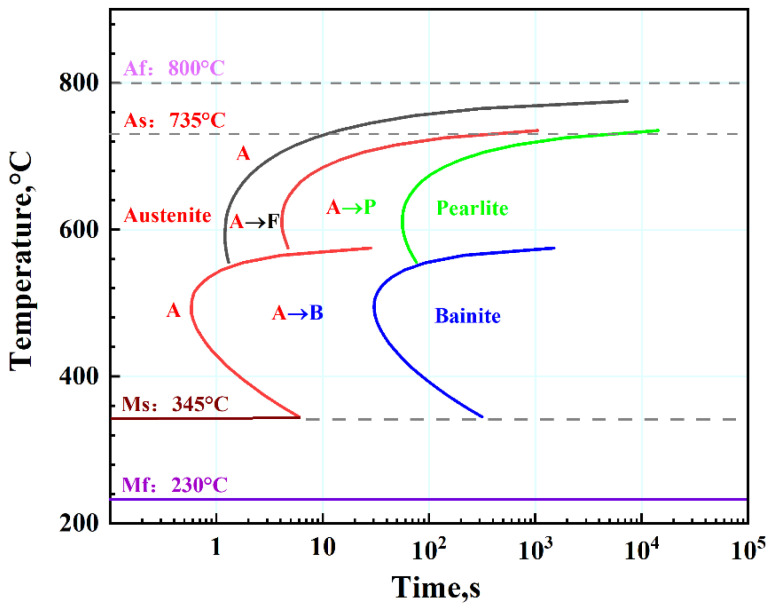
Temperature–time–transformation curves of 40Cr steel.

In order to accurately determine the kinetic parameters for phase transformation kinetic models, it is very important to correctly acquire the dilatometric properties of 40Cr during continuous cooling and its time temperature transformation (TTT) diagram. A cylindrical specimen for dilatometric test, 4 mm in diameter and 10 mm in length, is machined from the round bar to investigate continuous cooling transformations. The dilatometric test is performed in accordance with YB/T 5127-93 standard [19] using a dilatometer (DIL-805L, BÄHR-Thermoanalyse GmbH, Hüllhorst, Germany) to measure transformation strains during austenite decomposition under the continuous cooling conditions. The dilatometric specimen is firstly heated up to 1040 °C at a heating rate of 30 °C/s in vacuum and held for 5 min, and subsequently continuous cooling with a cooling rate of 30 °C/s. It must be emphasized that, due to the high alloy content of 40Cr, the martensitic finish temperature (Mf) is usually well below 0 °C. Therefore, when the temperature of specimen is lower than 100 °C, the liquid nitrogen is taken as a cooling medium so as to measure the martensitic finish temperature (Mf). Figure 3 shows the dilatometric curve of 40Cr specimen during continuous cooling. The dilatometric curve exhibits different slope changes, which can be attributed to different types of phase transformations occurred during continuous cooling. The transformation temperatures can be determined on the basis of the slope changes in dilatometric curve. According to the dilatometric curve obtained from dilatometric test, we can determine the martensitic start (Ms) and martensitic finish (Mf) temperatures, as well as the austenite start (As) and austenite finish (Af) temperatures, which are 345 °C, 230 °C, 735 °C, and 800 °C, respectively. In addition, combined the dilatometric test with the calculation using JMatPro^®^ 7.0 software [20,21], the time temperature transformation (TTT) diagram can also be obtained, as shown in Figure 4.

## 3. Mathematical Model

### 3.1. The Multi-Physics Field Coupling Model

Based on the metal–thermal–mechanical multi-physics field coupling framework, a macro-scale numerical model is established using the finite element method. The multi-physics field coupling model consists of temperature field model, microstructure field model, and stress–strain field model. The temperature field model is a crucial parameter in the coupling model, and the heat conduction equation [22,23] can be used to represent the transient heat conduction problem during phase transformation in quenching:

(1) $$\rho c\frac{\partial T}{\partial t}=\frac{\partial }{\partial x}(\lambda \frac{\partial T}{\partial x})+\frac{\partial }{\partial y}(\lambda \frac{\partial T}{\partial y})+\frac{\partial }{\partial z}(\lambda \frac{\partial T}{\partial z})+Q$$

In the equations, *x*, *y*, *z* represent the coordinate directions, *ρ* is the density, *c* is the specific heat capacity, *λ* is the thermal conductivity, *T*, *t* represents temperature and time, and *Q* is the internal heat source. If the initial condition assumes a uniformly distributed temperature in the specimen, the temperature function at *t* = 0 is given by

(2) $${\left.T(x,y,z)\right|}_{t=0}=T_{0}(x,y,z)$$

When the sample surface comes into contact with the cooling medium, the heat exchange conditions between them belong to the third type of mixed convection and radiation heat transfer boundary conditions, where thermal radiation can usually be neglected, yielding

(3) $${\left.-\lambda \frac{\partial T}{\partial n}\right|}_{\Gamma }=\bar{H}(T_{S}-T_{E})$$

In the equation, *n* represents the outward normal direction of the surface, $\bar{H}$ is the heat transfer coefficient, *T_S_* and *T_E_* are the temperatures of the sample surface and the medium, and *Γ* is the part surface.

The isothermal transformation curve is combined with the Scheil mode [24,25] to simulate the microstructurle evolution. The austenite transforms into ferrite, pearlite, and bainite, and the calculation is performed using the diffusion-controlled phase transformation JMAK equation [26,27]:

(4) $$\xi =1-\exp(-at^{n})$$

In the equation, *ξ* and *t* represent the transformation variable and time, while *a* and *n* represent the new phase nucleation and growth coefficients. The volume fraction of martensitic transformation is calculated using the modified non-diffusional phase transformation model by Inoue et al. [28,29], where the transformation variable is solely dependent on temperature, and the microstructure transformation variable can be expressed as

(5) $$\xi =1-\exp(\phi_{1}T+\phi_{2}(C-C_{0})+\phi_{31}\sigma_{m}+\phi_{32}\bar{\sigma }+\phi_{4})$$

In the equation, $\sigma_{m}$ and $\bar{\sigma }$ represent the average stress and equivalent stress, and *C* and *C*_0_ represent the carbon content. The isothermal transformation (temperature–time–transformation, TTT) curve can be used to determine $\phi_{1},\phi_{2},\phi_{31},\phi_{32},$ and $\phi_{4}$. Figure 4 shows the TTT curve for the experimental 40Cr steel, indicating a “C”-shaped isothermal transformation. Each isothermal transformation has an incubation period, and with decreasing temperature, both the transformation time and incubation time exhibit a decreasing-then-increasing trend.

The sum volume percentage of all microconstituents in the steel is 1. By determining the volume percentage of each constituent phase and the Vickers hardness of each constituent, it is possible to predict the Vickers hardness distribution state of the specimen after quenching. Then, with the conversion relationship between Vickers hardness and Rockwell hardness, the Rockwell hardness distribution state of quenched steel can be determined. Maynier et al. [30] suggest that the Vickers hardness of each constituent phase in steel is a function of steel composition and cooling rate. For the martensitic, bainitic, pearlitic, and ferritic models are, with the respective calculation formulas:

(6) $$H{\;V}_{M}=127+949C+27Si+11Mn+8Ni+16Cr+21\log V_{\gamma }$$

(7) $$\begin{array}{ll} H\;V_{B} & =323+185C+330Si+153Mn+65Ni+144Cr+191Mo\\ & +\left(89+53C-55Si-22Mn-10Ni-20Cr-33Mo\right)\log V_{\gamma } \end{array}$$

(8) $$HV_{F\text{-}P}=42+223C+53Si+30Mn+12.6Ni+7Cr+19Mo+(10\;-19Si+4Ni+8Cr+130V)\log V_{\gamma }$$

In the equation, *C*, *Si*, *Mn*, *Ni*, *Cr*, *Mo*, and *V* represent their respective mass percentages in the steel, with $V_{\gamma }$ indicating the cooling rate.

### 3.2. Finite Element Numerical Simulation

**Figure 5:**
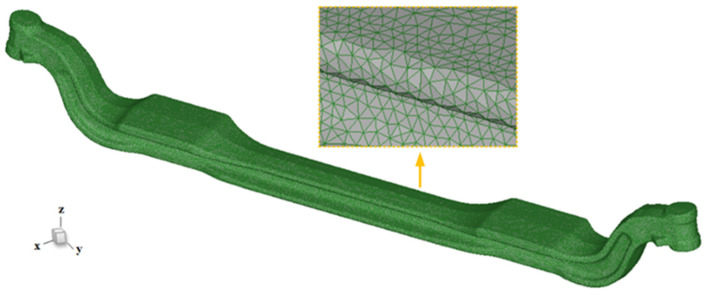
3D model of automobile front axle and meshing diagram of the front axle of the automobile.

In this numerical simulation of the automobile front axle, the research is based on four fundamental assumptions: firstly, assuming the front axle is a continuous solid material, uniformly and isotropically distributed in all directions; secondly, during the quenching process, the temperature of the quenching medium does not change; thirdly, the neglecting of the transfer time of quenching; and finally, all parts of the front axle undergo quenching at the same moment, with the same heat transfer coefficient; finally, both radiative heat transfer and latent heat of phase transformation are assumed to be 0 J/mol during quenching. Subsequently, the phase transition parameters, mechanical performance parameters, and thermal properties parameters of the 40Cr front axle are calculated using JMatPro^®^ software [20,21], which are given in Table 2. The front axle is divided into tetrahedral mesh with a total of 594,332 elements and 126,365 nodes, uniformly and appropriately distributed, as shown in Figure 5. All surfaces of the front axle are defined as heat transfer surfaces, and one end is fixed in the study to explore its deformation behavior more deeply. After completing the model definition, heat transfer coefficients are imported, and the temperature is set to rise from room temperature to 850 °C, followed by cooling in oil to room temperature. The heat transfer coefficients are shown in Figure 3 [31].

## 4. Results and Discussion

### 4.1. Microstructural Evolution

**Figure 6:**
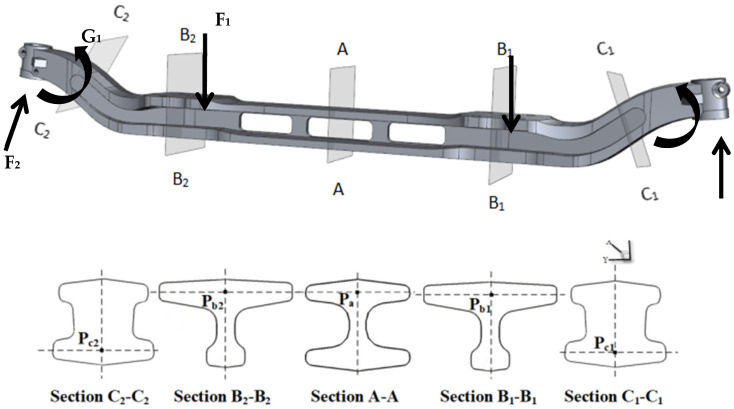
Force analysis of front axle and Schematic diagram of the temperature tracking point of the automobile front axle.

In terms of the usage conditions of the automobile front axle, it is mainly divided into vertical static load conditions, emergency braking conditions, and side slip conditions. The main force situations can be simplified as bending load F_1_ caused by the weight of the car, wheel reaction force F_2_, and torsional load G_1_ caused by braking [32]. Due to the symmetry of the automobile front axle, through the force analysis of the automobile front axle and combining with the work of Avikal et al. [5] who built a force analysis model for the front axle and used Ansys to conduct a stress analysis of the front axle of a heavy-duty truck to predict its service life, five representative vulnerable cross-sections of the front axle are selected for discussion: namely, the central cross-section A-A, 1/4 section B_1_-B_1_, edge region section C_1_-C_1_, 1/4 section B_2_-B_2_, and edge region section C_2_-C_2_. Tracking points P_c2_, P_b2_, P_a1_, P_b1_, and P_c1_ are selected from the sections, representing the farthest points from the surface, as shown in Figure 6.

**Figure 7:**
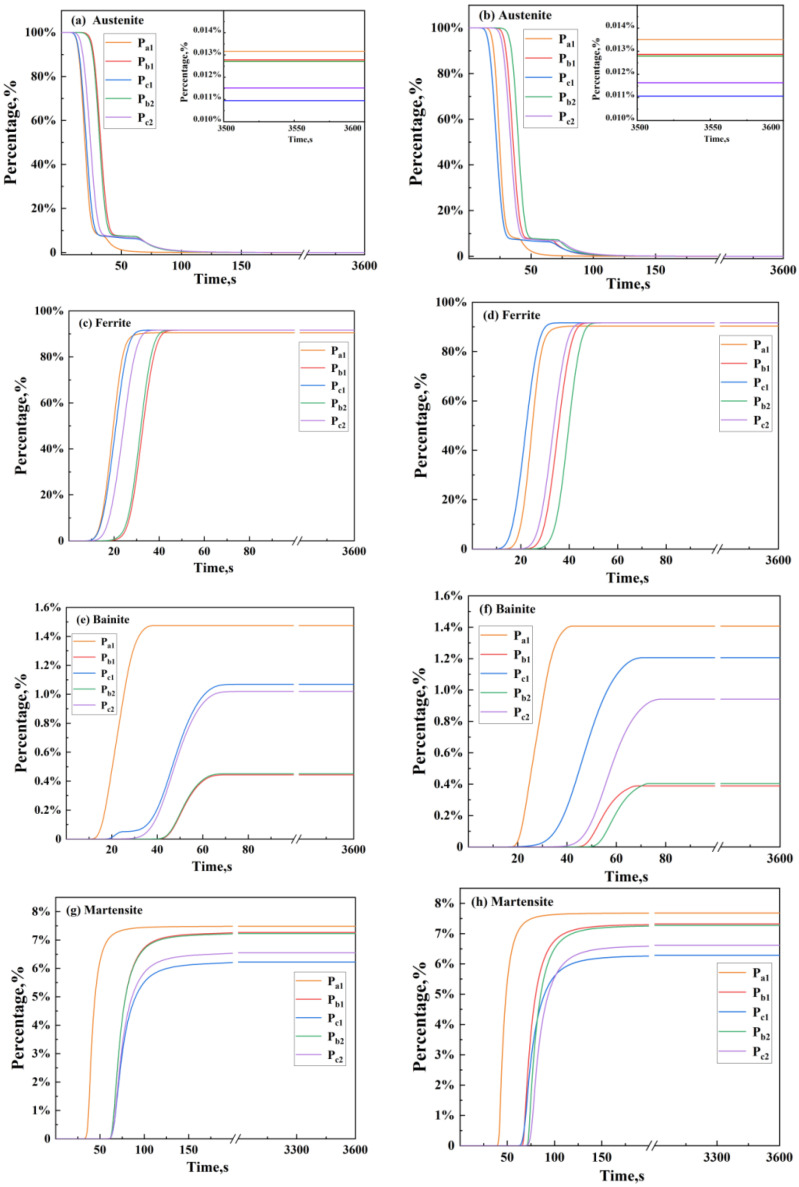
Microstructure evolution of the automobile front axle quenched by different immersion methods (**a**) horizontal, austenite (**b**) vertical, austenite (**c**) horizontal, ferrite (**d**) vertical, ferrite (**e**) horizontal, bainite (**f**) vertical, bainite (**g**) horizontal, martensite (**h**) vertical, martensite.

Figure 7a,c,e,g depict the evolution of austenite, ferrite, bainite, and martensite at five tracking points on the central cross-section A-A, 1/4 section B_1_-B_1_, edge region section C_1_-C_1_, 1/4 section B_2_-B_2_, and edge region section C_2_-C_2_ of the front axle during horizontal quenching. Figure 7b,d,f,h show the evolution of austenite, ferrite, bainite, and martensite at the five tracking points during vertical quenching. It can be observed from the figures that the same organization at the same tracking point changes over time in the same trend for both quenching methods, with only a slightly delayed onset of transformation in the case of vertical quenching. This indicates that the immersion method does not affect the transformation pattern of the core structure over time.

Overall, during the quenching process, the austenite almost completely transforms into constituents such as martensite, bainite, and ferrite. As shown in Figure 7a,b, at the five tracking points, the maximum residual austenite content during horizontal quenching is approximately 0.0131%, and during vertical quenching, it is approximately 0.0136%, which can be considered negligible. Figure 7c,d show that when the transformation of the structure approaches completion, the ferrite content at cross-section A-A during horizontal and vertical quenching is approximately 90.23% and 89.95%, at cross-section B-B it is approximately 91.47% and 90.95%, and at cross-section C-C it is approximately 91.59% and 91.31% for horizontal and vertical quenching, respectively.

Figure 7e–h show that when the structural transformation approaches completion, the sum of bainite and martensite content at cross-section A-A during horizontal and vertical quenching is approximately 8.96% and 9.06%, at cross-section B-B, it is approximately 7.72% and 7.84%, and at cross-section C-C, it is approximately 7.39% and 7.55% respectively. It can be observed that the transformation pattern is consistent between horizontal and vertical quenching, and the content of transformation products is similar. At cross-section C-C, the sum of bainite and martensite content is 1.57% lower than at cross-section A-A, accounting for approximately 21.25% of the sum of bainite and martensite content; and at cross-section C-C, it is 0.33% lower than at cross-section B-B, accounting for approximately 4.47% of the sum of bainite and martensite content. The experimental results are consistent with the numerical simulation results. Furthermore, from Figure 7e–h, it can be observed that the final content of martensite at cross-section C-C does not exceed 10%, making it the point with the lowest martensite content. This is mainly due to the presence of wall thickness differences, causing a slow temperature decrease during cooling. Before reaching the Ms point, the majority of the supercooled austenite transforms into ferrite. Since the ferrite content at cross-section C-C exceeds 90% and the martensite content is less than 10%, and martensite mainly determines the material’s hardness while ferrite, despite having good plasticity, lacks strength, cross-section C-C is considered a vulnerable point in the automobile front axle, and it should be tested as an important location during performance testing.

**Figure 8:**
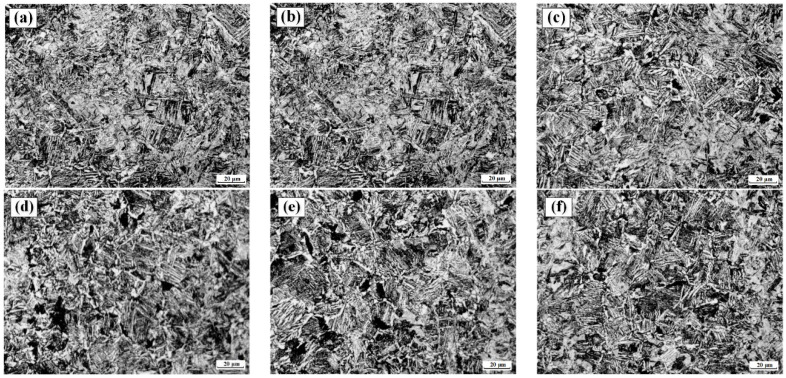
Microstructure of front axle after quenching under different immersion methods: (**a**) horizontal, a point; (**b**) horizontal, b point; (**c**) horizontal, c point; (**d**) vertical, a point; (**e**) vertical, b point; (**f**) vertical, c point.

Importantly, further analysis reveals that different quenching methods have a significant impact on the structural evolution of the automobile front axle. On the one hand, comparing Figure 7c,d, Figure 7e,f, and Figure 7g,h, it can be observed that when quenching vertically, the content of ferrite and martensite is slightly higher, while the content of bainite is slightly lower compared to horizontal quenching. However, the differences in content after quenching are still very small, indicating that the influence of the two quenching methods on the content of the central structure is minimal. On the other hand, since four of the selected tracking points are mutually symmetrical in the center of the front axle, it can be seen from the figure that when horizontal quenching is selected, the structural evolution curves of the symmetric tracking points are relatively consistent. However, when vertical quenching is chosen, the structural evolution of the five tracking points is noticeably uneven compared to horizontal quenching, indicating that the structural evolution of the entire front axle is more uniform when quenching horizontally than when quenching vertically. This is because horizontal quenching allows the entire front axle to quickly enter the cooling medium, ensuring that all parts of the front axle start cooling and undergoing structural transformation simultaneously. In contrast, vertical quenching, due to the large size of the front axle, prevents all parts from cooling simultaneously, resulting in differences in the time of structural transformation and extremely uneven temperature distribution. By comparing Figure 8a,d, Figure 8b,e, and Figure 8c,f, it can be observed that the structures experimentally obtained by quenching the automobile front axle in different ways are basically the same. However, the uniformity of the structure obtained by horizontal quenching is qualitatively slightly better than that obtained by vertical quenching. After cooling in the cooling medium by vertical quenching, the structure contains slightly more ferrite, which leads to a slightly lower yield strength of the front axle. The experimental results are consistent with the numerical simulation results.

**Figure 9:**
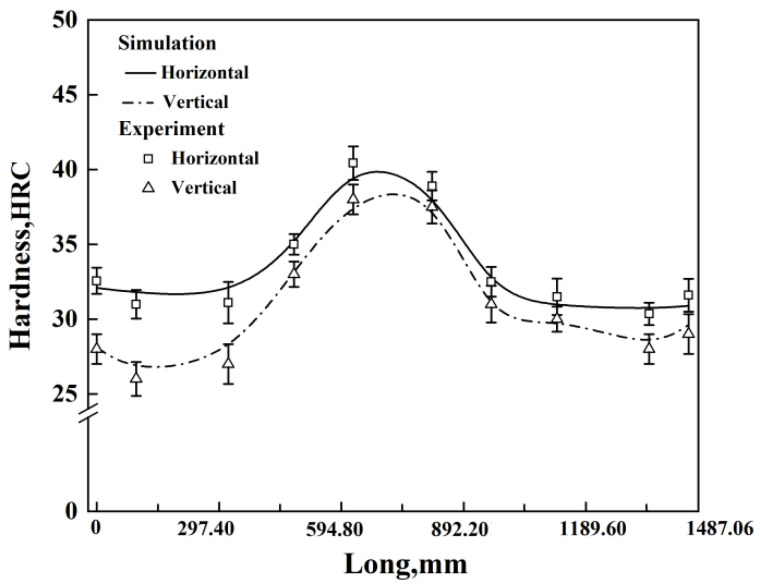
Hardness of front axle after quenching under different immersion methods.

Figure 9 illustrates the distribution pattern of the average hardness of the automobile front axle after quenching with different immersion methods. From the figure, it can be seen that, within the allowable error range, the simulation results of the hardness distribution of the front axle are basically consistent with the experimental results. Observing the overall hardness distribution graph of the front axle after quenching with different immersion methods, it can be seen that the general distribution pattern of hardness is similar. Due to the thinner middle part of the front axle, with higher heat transfer efficiency, the percentage content of martensite and bainite is higher, resulting in higher hardness in the middle part than on both sides, which is consistent with the above structural analysis.

In the graph, it can be observed that when entering the cooling medium in a horizontal manner, the maximum hardness is approximately 39.7 HRC, and the minimum hardness is about 31.5 HRC, which is a hardness difference of about 8.2 HRC. When entering the cooling medium in a vertical direction, the maximum hardness is about 37.5 HRC, and the minimum hardness in the near-edge region is about 27.2 HRC, which is a hardness difference of about 10.3 HRC. It can be seen that the hardness obtained when entering the cooling medium vertically is slightly lower overall than the hardness obtained when entering the cooling medium horizontally, and the hardness distribution is more uneven. Moreover, observing the hardness distribution after vertical quenching, it can be found that the hardness values in the rear part of the front axle entering the cooling medium are generally slightly lower than those in the front part entering the cooling medium. This is because the heat flow will rise around the front axle, and the cooling rate of the part entering the cooling medium later will decrease, affecting the transformation of martensite.

### 4.2. Deformation Behavior

Among the coefficients of thermal expansion, the linear expansion coefficient is the most commonly used indicator for deformation calculations. It represents the relative elongation of an object when the temperature increases by 1 °C. When the temperature rises from *T*_1_ to *T*_2_, and the length of an object in a certain direction increases from *L*_1_ to *L*_2_, the average linear expansion coefficient α of the material within this temperature range is given by

(9) $$\alpha =\frac{L_{2}-L_{1}}{L_{1}(T_{2}-T_{1})}=\frac{∆L}{L_{1}(∆T)}$$

where ∆*L* represents the increase in length of the sample from *T*_1_ to *T*_2_, cm, and ∆*T* represents the increase in temperature, °C. If the length (*L*) of the material is recorded at each temperature (*T*) and a *T*-*L* curve is plotted, for any chosen temperature point (t) on the curve, the instantaneous coefficient of thermal expansion of the material at that point is given by the slope $\frac{dL}{dT}$ of the curve:

(10) $$\alpha_{t}=\frac{1}{L}\cdot \frac{dL}{dT}$$

**Figure 10:**
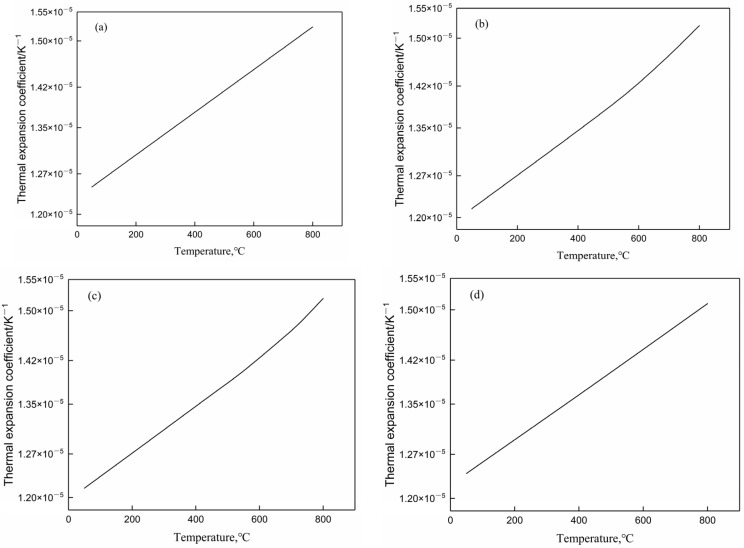
Thermal expansion coefficient of each structure during quenching: (**a**) ferrite; (**b**) pearlite; (**c**) bainite; (**d**) martensite.

The thermal expansion coefficients of various constituents in the simulated calculation of the automobile front axle are shown in Figure 10. It is evident from Figure 10a–d that at 800 °C, the thermal expansion coefficients of various structures are all between 1.50 × 10^−5^–1.55 × 10^−5^/K^−1^, with little difference. When the temperature is near room temperature, the thermal expansion coefficients of ferrite and martensite are greater than those of pearlite and bainite.

In order to further understand the influence of using different quenching methods on the deformation behavior of the front axle and to verify the reliability of the simulation results, the height and width changes in five representative sections of the front axle were studied. These sections include the front axle center section A-A, 1/4 section B_1_-B_1_, edge region section C_1_-C_1_, 1/4 section B_2_-B_2_, and edge region section C_2_-C_2_. Three-dimensional scanning was performed using a 3D scanner to measure the post-quenching 3D dimensions of the front axle, and the deformation values for the representative sections were obtained. The obtained results were then compared and verified against the predicted deformation values from the simulation.

**Figure 11:**
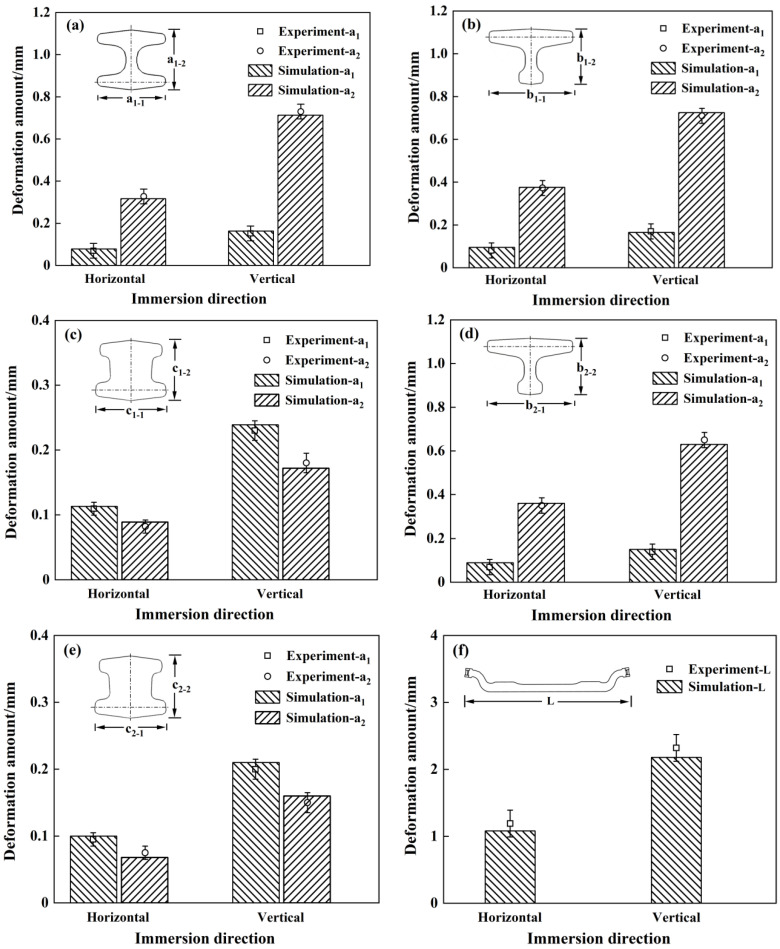
Deformation of section and total length of front axle after quenching with different immersion methods: (**a**) section A-A; (**b**) section B_1_-B_1;_ (**c**) section C_1_-C_1;_ (**d**) section B_2_-B_2;_ (**e**) section C_2_-C_2;_ (**f**) total length.

Figure 11a–e respectively represent the deformation values and experimental results of five selected sections of the front axle of the automobile: namely, the front axle center section A-A, 1/4 section B_1_-B_1_, edge region section C_1_-C_1_, 1/4 section B_2_-B_2_, and edge region section C_2_-C_2_. The deformation values of each section were compared for different immersion methods. Figure 11f provides a comparison of the total length deformation of the front axle after quenching using different immersion methods. The impact of different immersion methods on the deformation of the automobile front axle varies, and the effects on vertical and horizontal deformation of the cross-sections under the same immersion method are also distinct. Overall, the deformation under horizontal quenching is less than that under vertical quenching. For the A-A cross-section, the maximum experimental deformation in the vertical direction is approximately 0.73 mm, while the numerical simulation yields a maximum deformation of about 0.73 mm. In the horizontal direction, the maximum experimental deformation is around 0.32 mm, and the numerical simulation shows a maximum deformation of about 0.31 mm, with both occurring in the vertical direction of the representative cross-section. Concerning the B_1_-B_1_ cross-section, the deformation patterns in the horizontal and vertical directions are similar to those of the A-A cross-section. The maximum experimental deformation in the vertical direction is approximately 0.71 mm, and the numerical simulation indicates a similar maximum deformation of about 0.71 mm. In the horizontal direction, the maximum experimental deformation is roughly 0.37 mm, while the numerical simulation predicts a maximum deformation of about 0.37 mm. As for the C_1_-C_1_ cross-section, the deformation is relatively smaller compared to the other two representative cross-sections. The maximum experimental deformation in the vertical direction is about 0.22 mm, and the numerical simulation yields a maximum deformation of around 0.24 mm. In the horizontal direction, the maximum experimental deformation is approximately 0.11 mm, and the numerical simulation predicts a maximum deformation of about 0.11 mm, with the maximum deformation occurring in the horizontal direction, unlike the A-A and B_1_-B_1_ cross-sections. The deformation patterns of the B_2_-B_2_ and C_2_-C_2_ cross-sections are similar to those of the B_1_-B_1_ and C_1_-C_1_ cross-sections, but with smaller deformations in both horizontal and vertical directions compared to the B_1_-B_1_ and C_1_-C_1_ cross-sections. Regarding the total length change, the experimental deformation is about 1.18 mm under horizontal quenching, while the numerical simulation predicts a deformation of about 1.07 mm. Under vertical quenching, the experimental deformation is approximately 2.31 mm, and the numerical simulation forecasts a deformation of about 2.18 mm, exceeding the deformation under horizontal quenching by twice the amount. Therefore, it can be concluded that the deformation under vertical quenching is greater than that under horizontal quenching.

At the same time, the figures show that the numerical simulation results of the deformation for the five sections and the total length generally match the experimental results, indicating that the numerical results are reliable within the allowed error. In Figure 11a–e, it is observed that different immersion methods have an impact on the deformation of the front axle, and there are differences in the deformation effects in the vertical and horizontal directions for the same immersion method. After horizontal quenching, the average experimental deformation and average numerical simulation deformation for the five representative sections of the front axle are approximately 0.16 mm and 0.17 mm, respectively, while after vertical quenching, they are approximately 0.31 mm and 0.33 mm, respectively, differing by about two times. There are also some differences in the deformation of the regions where the front axle enters the cooling medium later. The region entering the cooling medium later experiences slightly smaller deformation compared to the region entering the cooling medium earlier, which is attributed to the slower heat transfer rate in the region entering later due to the heat flow generated by the region entering earlier. From Figure 11f, it can be seen that the experimental deformation and numerical simulation deformation of the total length of the front axle after horizontal quenching are approximately 1.18 mm and 1.07 mm, respectively, both smaller than the experimental deformation of approximately 2.31 mm and numerical simulation deformation of approximately 2.18 mm after vertical quenching.

## 5. Conclusions

(a) Attributed to the influence of the different shapes and dimensions of various regions on the quenching microstructure evolution, the front axle exhibits a decrease in bainite and martensite structures and an increase in ferrite along the length direction from the center to the edge regions. Specifically, during horizontal quenching, the sum of bainite and martensite content at section C-C is approximately 7.39%. In the cross-sectional comparison between the edge region C-C and the center A-A of the front axle, the combined content of bainite and martensite in section C-C is 1.57% lower, representing a reduction of approximately 21.25% compared to the total content of bainite and martensite. Similarly, in the comparison between section C-C and section B-B, the combined content of bainite and martensite in section C-C is 0.33% lower, corresponding to a decrease of about 4.47% relative to the total content of bainite and martensite. The experimental results align with the numerical simulation outcomes.
(b) When adopting the vertical quenching method, the larger dimensions of the front axle result in uneven cooling, leading to differences in the transformation time and an extremely uneven temperature distribution. Both experimental results and numerical simulations indicate that the central part of the front axle attains the maximum hardness of approximately 37.5 HRC, while the near-edge region achieves the minimum hardness of about 27.2 HRC, resulting in a hardness difference of around 10.3 HRC. In contrast, when using the horizontal quenching method, the hardness difference among different regions of the front axle is approximately 8.2 HRC. Therefore, considering the uniformity of hardness, the horizontal immersion quenching method is preferable.
(c) On the one hand, the sequential immersion of different regions of the front axle into the quenching medium causes variations in heat flow and affects the heat transfer rate. On the other hand, the differences in the content of martensitic structures obtained in different regions of the front axle result in variations in the magnitude of deformation during the phase transformation process. This leads to a decreasing trend in deformation along the length of the front axle from the center to the edge. The numerical simulation results indicate that, with the vertical quenching method, the deformation of the central cross-section A-A, 1/4 cross-section B-B, edge region cross-section C-C, and the length direction are approximately 0.73 mm, 0.71 mm, 0.24 mm, and 2.31 mm, respectively, with an error less than 3% compared to the actual measured results.
(d) Horizontal and vertical quenching methods result in experimental deformations of approximately 0.32 mm and 0.73 mm, respectively, at the central cross-section A-A of the front axle. The experimental deformation of the total length of the front axle is approximately 1.18 mm for horizontal quenching and 2.31 mm for vertical quenching. Therefore, choosing the horizontal immersion method for quenching, as opposed to the vertical method, reduces the deformation by approximately 56.2% and 48.9% on the representative cross-section A-A and the total length of the front axle, respectively, resulting in an overall reduction in deformation.

## Figures and Tables

**Table 1:** Composition analysis of 40Cr steel (mass fraction %).

Steel	C	Si	Mn	Cr	Ni	Cu	Al	Ti	P	S	Fe
40Cr	0.4	0.21	0.64	0.95	0.02	0.03	0.024	0.008	0.021	0.05	Bal.

**Table 2:** Thermal conductivity, heat capacity, and Young’s modulus of 40Cr steel.

Temperature, °C	Thermal Conductivity, W/m °C	Heat Capacity, J/kg °C	Young’s Modulus, GPa
20	51.15	457.25	210.89
100	49.73	489.40	207.60
200	47.10	526.42	201.86
300	44.01	571.95	194.06
400	40.53	632.95	184.18
500	37.05	709.21	172.38
600	33.86	816.33	159.01
700	31.57	956.46	144.72
800	26.70	599.04	127.61
900	27.89	614.53	117.87
1000	29.07	630.12	108.01

## Data Availability

The original contributions presented in the study are included in the article, further inquiries can be directed to the corresponding author.
